# Vascular injuries after minor blunt upper extremity trauma: pitfalls in the recognition and diagnosis of potential "near miss" injuries

**DOI:** 10.1186/1757-7241-16-16

**Published:** 2008-11-25

**Authors:** Jonathan T Bravman, Kyros Ipaktchi, Walter L Biffl, Philip F Stahel

**Affiliations:** 1 Department of Orthopaedic Surgery, Denver Health Medical Center, University of Colorado School of Medicine, 777 Bannock Street, Denver, CO 80204, USA; 2 Department of Surgery, Denver Health Medical Center, University of Colorado School of Medicine, 777 Bannock Street, Denver, CO 80204, USA

## Abstract

**Background:**

Low energy trauma to the upper extremity is rarely associated with a significant vascular injury. Due to the low incidence, a high level of suspicion combined with appropriate diagnostic algorithms are mandatory for early recognition and timely management of these potentially detrimental injuries.

**Methods:**

Review of the pertinent literature, supported by the presentation of two representative "near miss" case examples.

**Results:**

A major diagnostic pitfall is represented by the insidious presentation of significant upper extremity arterial injuries with intact pulses and normal capillary refill distal to the injury site, due to collateral perfusion. Thus, severe vascular injuries may easily be missed or neglected at the upper extremity, leading to a long-term adverse outcome with the potential need for a surgical amputation.

**Conclusion:**

The present review article provides an outline of the diagnostic challenges related to these rare vascular injuries and emphasizes the necessity for a high level of suspicion, even in the absence of a significant penetrating or high-velocity trauma mechanism.

## Background

Upper extremity arterial injuries secondary to minor, non-penetrating trauma mechanisms, such as low energy traumatic joint dislocations, are very rare. In a study of 1,565 upper extremity dislocations, arterial lesions were detected in 0.97% and 0.47% of all cases with closed shoulder or elbow dislocations, respectively [1]. Interestingly, this rare entity has been first described in the French literature almost 100 years ago, and was found to be associated with a high mortality due to delayed recognition and a lack of effective treatment strategies [2]. Elderly patients appear to be particularly susceptible to vascular injuries due to loss of arterial elasticity [3]. In this article, we outline the diagnostic challenges related to these rare vascular injuries, based on the description of two clinical "near miss" cases, and provide an updated review of the pertinent literature.

## "Near miss" case #1

**Figure 1:**
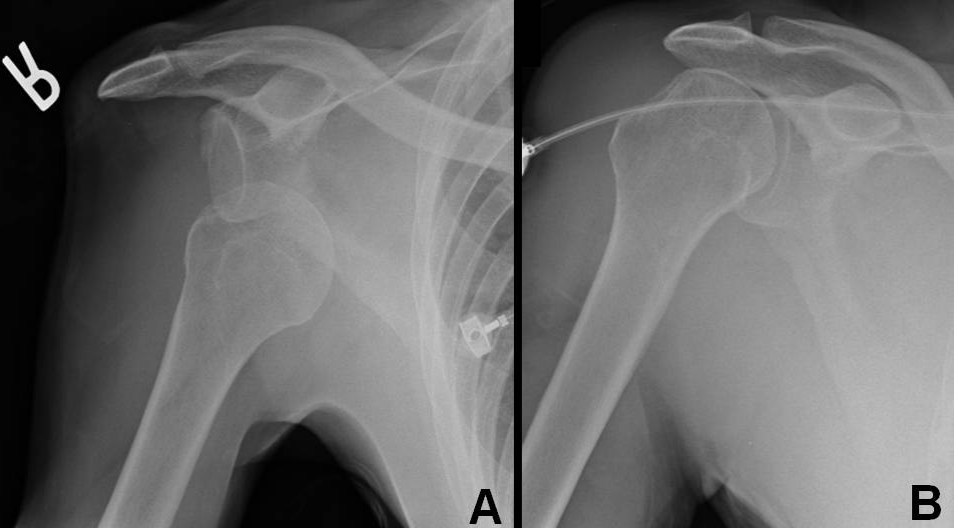
Case demonstration of a 46-year-old right hand dominant male who sustained a fall from standing onto the right shoulder. Injury X-ray demonstrates an anterior shoulder dislocation (panel A) which was successfully reduced in the emergency department (panel B).

**Figure 2:**
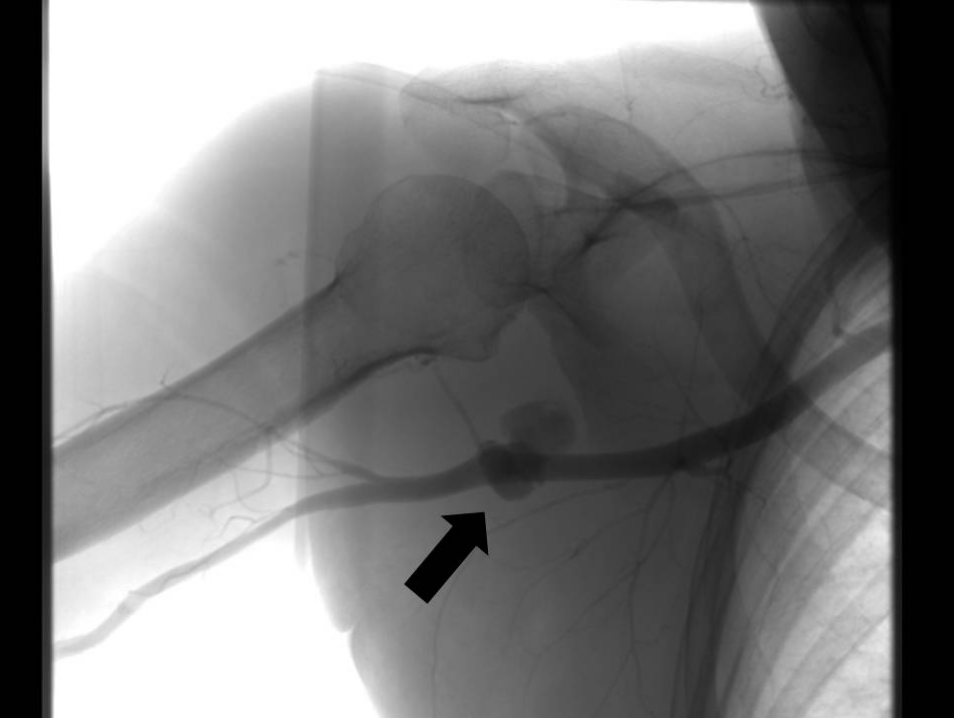
Same case as in figure 1. Angiography demonstrates a traumatic axillary artery pseudoaneurysm (arrow).

A 46 year-old right hand dominant male presented to our emergency department (ED) after sustaining a fall from standing height off a curb while intoxicated. The patient had a history of recurrent right shoulder dislocations after low-energy mechanisms. He has been previously treated conservatively at outside facilities, with closed reduction and temporary immobilization. On current presentation to the ED, the patient complained of right shoulder pain. Clinically, he had a typical deformity consistent with an anterior shoulder dislocation. This diagnosis was confirmed by plain x-rays (Fig. 1A). Neurovascular exam demonstrated normal (2+) and symmetric radial pulses with 5/5 motor function and fully intact light touch sensation and 2-point discrimination in the distribution of the radial, median, and ulnar nerves. Conscious sedation was administered and closed reduction was carried out uneventfully in the ED, by a Kocher maneuver (Fig. 1B). The post-reduction exam revealed an unchanged neurovascular exam, and the plan was made for patient discharge with elective follow-up in orthopedic clinic. Shortly before discharge, the new finding of a "pectoral swelling" was noted. At this time, the orthopedic surgery service was consulted for concern of a potential pectoralis muscle rupture. Upon the repeat evaluation by the orthopedic team, an expanding hematoma was appreciated in the axilla and anterolateral chest wall. Additionally, compared to the previously documented exam, a change in neurologic status was noted with a decrease in light touch sensation in the distribution of the median and ulnar nerve, while motor function remained intact. In addition, the right arm was found to have a mildly hyperemic appearance. Distal pulses were present and palpable, and the arm-arm arterial index was measured to be 1.1 (i.e. ipsilateral blood pressure compared to the contralateral side). Based on the neurological finding of a *"soft sign" *for arterial injury (Table 1), a concern for arterial injury was raised, and a further diagnostic work-up was initiated. An emergent angiography was performed, which revealed a leaking pseudoaneurysm of the right axillary artery (Fig 2, arrow). The vascular surgery team was consulted and the patient was immediately taken to the operating room. The surgical exploration revealed an avulsion injury to two posterior and inferiorly directed branches from the base of the circumflex humeral artery with extension into the axillary artery. These branches were ligated and a primary repair of the remaining axillary artery defect was performed. Forearm fasciotomies were performed to prevent an ischemia/reperfusion-mediated compartment syndrome. The vascular repair was successful, and the patient recovered well secondary to a repeat return to the OR for wound closure of the fasciotomy incisions. On final follow-up in orthopedic clinic, the patient had an intact neurologic exam and well perfused right arm, with strong palpable symmetric radial pulses.

**Table 1:** Clinical signs for prediction of an arterial extremity injury.

***"Hard signs"***	***"Soft signs"***
Active or pulsatile hemorrhage	Asymmetric extremity blood pressures
Pulsatile or expanding hematoma	Stable and non-pulsatile hematoma
Clinical signs of limb ischemia	Proximity of wound to a major vessel
Diminished or absent pulses	Peripheral neurological deficit
Bruit or thrill, suggesting AV-fistula	Presence of shock/hypotension

The presence of a *"hard sign" *of an arterial injury warrants an immediate surgical exploration with the option of an on-table angiography. In contrast, the *"soft signs" *are less specific in predicting a significant arterial extremity injury. In exclusive presence of a *"soft sign"*, such as an asymmetric ankle-brachial-index, the recommended further diagnostic workup includes an angiography or CT-angiography.

## "Near miss" case #2

**Figure 3:**
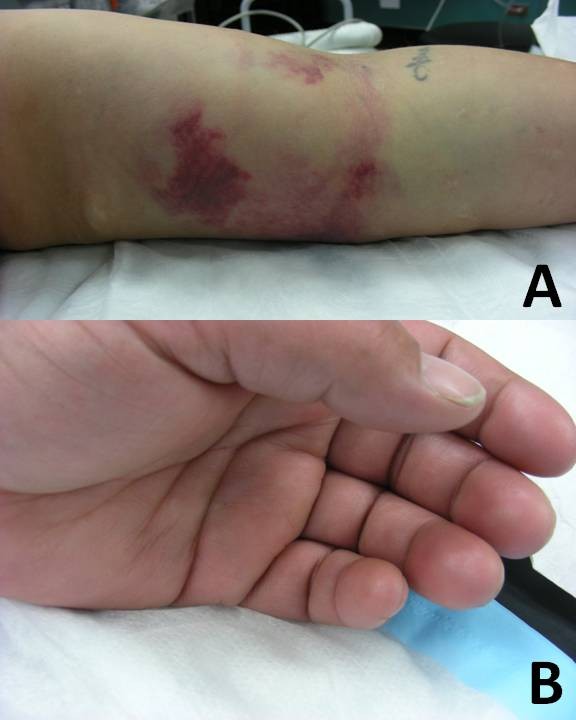
Case demonstration of a 38-year-old right hand dominant woman who sustained a twisting injury to her left arm during an assault. Clinically, her left elbow showed ecchymosis on the medial side (panel A). Her ipsilaterla hand remained well perfused (panel B), with a strong radialis pulse and a normal capillary refill, despite the presence of a significant arterial injury.

**Figure 4:**
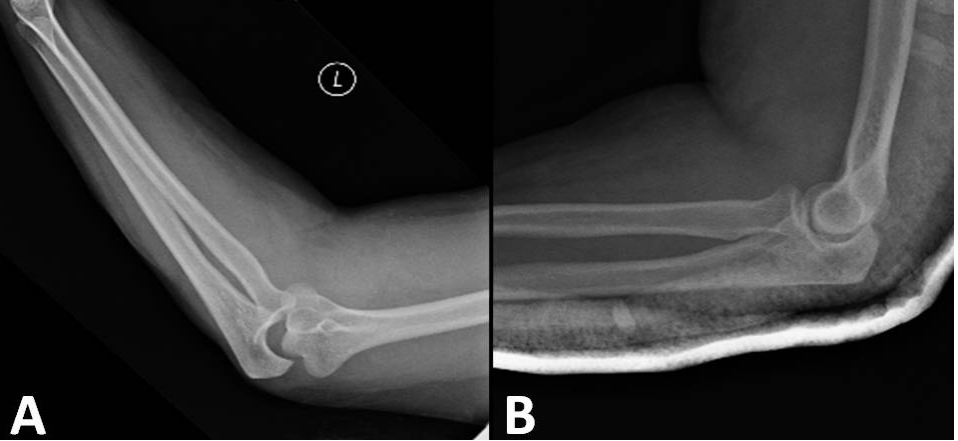
Same case as in figure 3. X-rays before (panel A) and after (panel B) closed reduction of a left elbow dislocation.

A 38 year-old right hand dominant woman presented to the ED intoxicated, one day after being assaulted. Per the patient's report, her left arm had been twisted during the altercation. She complained of left arm pain. Her past medical history was relevant for chronic alcohol abuse. On first evaluation, the left arm appeared cooler than the right arm, and there was a gross deformity and instability at the elbow joint with an ecchymotic area over the medial side of the elbow (Fig 3A). Clinically, the patient had a normal perfusion in her left hand (Fig. 3B). Her neurologic evaluation revealed an intact 5/5 motor function of the left hand and intact sensation to light touch and 2-point discrimination. The radial pulse was symmetrically palpable (2+). The patient was radiographically found to have a traumatic elbow dislocation (Fig. 4A), which was successfully reduced upon first attempt under conscious sedation in the ED (Fig. 4B).

**Figure 5:**
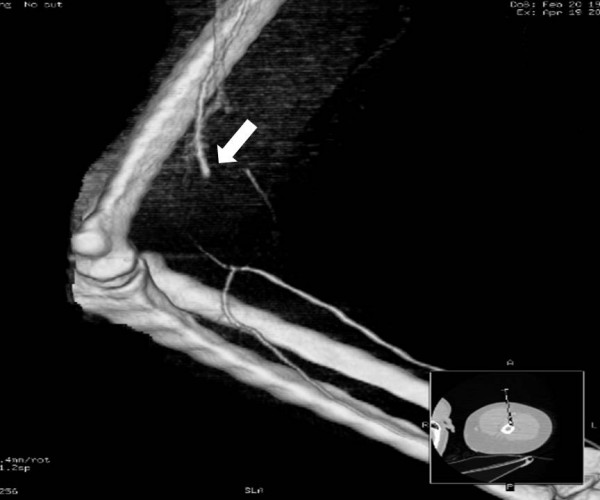
Same case as in figures 3, 4. CT-angiography with 3D-reconstruction demonstrating a 4 to 5 cm long brachial artery laceration at the elbow (arrow) with reconstitution of forearm vessel flow via collateral perfusion.

**Figure 6:**
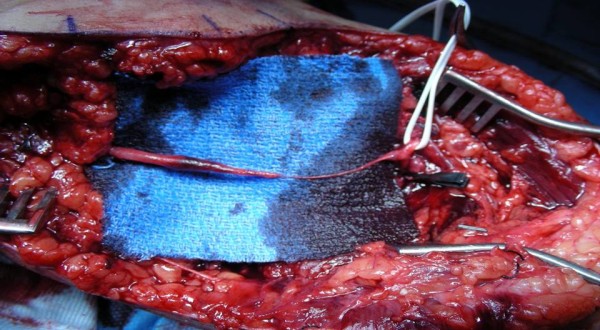
Same case as in figures 3, 4, 5. The intraoperative finding correlates to preoperative CT-angiography, revealing a brachial artery traction injury of about 5 cm length, located proximal to the bifurcation.

After closed reduction, the radial artery pulse was faintly palpable, while Doppler signals were robust. The arm-arm arterial index was found to be pathological, with a value of 0.7. The patrient's left hand continued to appear well perfused with a recapillarisation time of <2 seconds. A CT-angiography was ordered with concern for arterial injury and revealed an abrupted contrast flow at the level of the elbow, with collateral reconstitution of vascular flow in the forearm (Fig. 5, arrow). The patient was taken emergently to the OR for exploration of the antecubital fossa, revealing a traction-avulsion injury of 5 cm length at the level of the brachial artery proximal to the bifurcation (Fig. 6). In addition, the anterior joint capsule was found to be completely torn. Arterial repair was performed by interpositional reverse saphenous vein grafting, which re-established a palpable radial artery pulse. Given the absence of forearm ischemia prior to the repair, as well as soft forearm compartments intraoperatively, the decision was made not to perform a forearm fasciotomy. The arm was placed in a posterior splint until the wound was healed, and the patient had an uneventful postoperative course at follow-up in orthopedic clinic.

## Discussion

Traumatic injuries to the axillary and brachial arteries remain rare, representing 15–20% of arterial injuries to the upper limb [4]. Approximately 6% of these injuries are attributable to blunt trauma, with the majority occurring in the setting of fracture-dislocations. Less than 1% of vascular injuries to the upper extremity are associated with a traumatic dislocation alone [1]. Elderly patients appear more susceptible to vascular injuries, with the majority of reported cases occurring in patients over the age of 50 [3]. Interestingly, up to a third of all patients have a history of previous joint dislocations, suggesting arterial incarceration in scar tissue, which may render the vessel more susceptible to injury during a subsequent dislocation [5].

Several potential mechanisms relating to the particular regional anatomy have been postulated which may account for upper extremity vascular injuries. The axillary artery is typically divided into three segments relative to its relationship with the pectoralis minor muscle. The third segment – defined as the portion distal to the lower edge of the pectoralis minor – appears to be most frequently injured (86%) [6]. Adoriasio [4] and Milton [7] independently proposed a mechanism by which the axillary artery is exposed to direct injury by the dislocating humeral head, given its relatively fixed anatomical position between the subscapular and humeral circumflex arteries. Multiple authors have additionally proposed a mechanism by which the pectoralis minor muscle acts as fulcrum for the artery, thus enabling a vascular injury by kinking, shearing or compression [3,6,8,9]. Although axillary artery injuries are fairly common, fewer than 50 cases related to anterior shoulder dislocations have been reported in the literature, to our knowledge [3,4,10-20].

A major contributing factor for brachial artery injuries at the elbow region is related to a vascular entrapment underneath the lacertus fibrosus in the antecubital fossa. This anatomical relation explains the high incidence of brachial artery injuries proximal to the bifurcation, due to the relative immobility of the artery which prohibits a longitudinal excursion to compensate for forearm rotation about the elbow secondary to elbow dislocations and distal humerus fractures [21]. The elbow has a circumferential "network" of collaterals which feed the radial and ulnar recurrent and interosseus vessels, even in the absence of brachial artery flow. This circumstance explains the relative success of the historical practice of ligation of brachial and/or radial and ulnar arteries in elbow dislocations performed to control posttraumatic bleeding intraoperatively [22].

A pathognomonic "triad" has been described to diagnose vascular lesions in shoulder dislocations, consisting of anterior shoulder dislocation, expanding axillary hematoma and diminished peripheral pulse [3]. Similarly, in closed elbow dislocations, the absence of a radial pulse has been noted to be main predicting factor of an arterial injury [23]. As outlined by the two representative cases in the present paper, the reliance on peripheral pulses alone can be misleading. Sparks *et al. *described 30 patients with absent peripheral pulses and clinical signs of ischemia, of which only 12 cases were found to have arterial injuries by angiography [1]. On the other hand, palpable distal pulses or pulses detected by Doppler ultrasound may be present even in the instance of complete arterial disruption, due to abundant collateral flow [24,25]. Therefore, the physical exam alone is generally regarded as inadequate for diagnosis of peripheral vascular injury in extremity trauma and has been shown to be a poor predictor of arterial injuries [26-28]. In fact, the "classic" signs of arterial insufficiency may be absent in up to 40% of patients with upper extremity joint dislocations [1,29]. Clinical signs of arterial injury have been stratified based on their predictive value into "hard" and "soft" signs (table 1). The presence of a "hard" sign of vascular injury mandates an immediate surgical exploration and vascular repair [26,30-32]. In contrast, clinical "soft" signs (table 1) are much less specific in the diagnosis of a significant arterial injury, and have been found to lack an adequate predictive value [26,33].

Management strategies for patients with vascular injuries have gradually changed over time. During wartimes, a protocol of operative exploration was advocated based on the proximity of injury alone. This concept was abandoned when it became apparent that such a paradigm was not transferable to low-velocity civilian injuries due a low efficiency [32,34,35]. In the 1970's and 80's, arteriography became the "gold standard", and was abandoned more recently, due to invasive risks of iatrogenic complications and the observation that the angiographic screening rarely led to a change in patient managemen [33,34,36-41]. Doppler ultrasound was demonstrated to be an effective diagnostic tool, albeit its sensitivity being highly operator-dependent [30,42,43]. More recently, the "arterial pressure index" (API) – also described as the "ankle-brachial index" (ABI) or the "ankle-arm index" (AAI) – has become a new standard as a screening tool in the potentially vascular injured limb [39,41]. The API is performed by placing a blood pressure cuff just above the ankle or wrist of the injured limb and the systolic pressure is determined by Doppler probe at the respective dorsalis pedis or radial artery. Identical measurement is performed on an uninjured limb and the API is calculated by dividing the systolic pressure in the injured limb by the systolic pressure in the uninjured limb. This tool has been validated in the setting of penetrating and blunt extremity injuries [39,41,44]. An API value of < 0.9 was found to have a sensitivity of 95% and specificity of 97% for a major arterial extremity injury [41]. A different study on blunt orthopaedic extremity injuries described the negative predictive value of 100% for an API > 0.9 to exclude an arterial injury [44]. In the present paper, only one of two cases had a pathological API of 0.7, while the first patient described in this report here had a misleading API of 1.1, suggesting the absence of an arterial injury. In recent years, CT-angiography (CT-A) has come to play an increasing role in diagnosing suspected peripheral artery injuries. Compared to traditional angiography, as the previous "gold standard", modern CT-A using multislice fine resolution technique has been shown to be less invasive, while yielding a similar diagnostic sensitivity and a more widespread availabilty in the acute workup of trauma patients [45].

Multiple treatment options have been described for arterial injuries associated with extremity trauma. Case reports describe successful outcomes using endovascular techniques employing thrombolysis and stent placement [13]. However, most authors agree that the most adequate treatment modality remains in the surgical exploration with the intraoperative option of a thrombectomy, end-to-end anastamosis, saphenous vein graft or prosthetic allograft, with ligation of avulsed collaterals [1]. Prophylactic forearm fasciotomies should be performed in all ischemic limbs due to the high risk of postoperative compartment syndrome secondary to reconstitution of arterial flow, leading to ischemia/reperfusion syndrome.

Unfortunately, until present, there is no established or putative algorithm available which may allow the straight-forward clinical work-up for discrimination and identification of those rare patients who sustained a significant vascular injury after minor blunt upper extremity trauma. Obviously, a full work-up by CT-angiography for every single patient presenting to the emergency department for a traumatic shoulder or elbow dislocation is neither feasible, nor cost-effective. Thus, the emergency physician in charge of these patients in the first place must have a high level of suspicion, in conjunction with the knowledge on the "hard signs" of vascular injuries, as outlined in table 1. While common clinical knowledge implies that a significant vascular injury may require the history of either a high-energy blunt trauma, or a penetrating trauma mechanism, as a prerequisite, we clearly dismiss this notion in the present paper. Key to success is the awareness of potentially detrimental vascular injuries in minor upper extremity trauma, combined with the knowledge of "hard clinical signs" of vascular injury which will mandate immediate further work-up, with a high likelihood of the need for surgical intervention.

## Conclusion

Vascular injuries in low energy trauma are rare and are easily missed. A high level of suspicion, in conjunction with the knowledge of sensitive and specific clinical signs, is paramount for an accurate and timely diagnosis. A thorough physical exam, including determination of the API, is crucial in the early assessment of a patient with concern for a vascular extremity injury. Based on the presence or absence of "hard" clinical signs of arterial injury, an early indication must be placed for immediate surgical exploration versus additional diagnostic interventions, such as an arteriography or CT-A. In contrast, simple observation represents the prerequisite for a detrimental outcome, since time is of the essence in recognition and management of these rare injuries with a potential for high morbidity and mortality.

## Competing interests

The authors declare that they have no competing interests.

## Authors' contributions

JTB and KI wrote the first draft of the manuscript. WLB and PFS revised the manuscript and performed the final editing. KI and PFS provided the two clinical case examples. All authors read and approved the final version of the manuscript.

## Consent

Written informed consent was obtained by the two patients presented in  this paper for publication of their individual case reports.
